# Developing a brief older adults' physical activity questionnaire

**DOI:** 10.1111/ggi.14986

**Published:** 2024-09-30

**Authors:** Koutatsu Nagai, Ryota Matsuzawa, Hiroyuki Sasai, Kayoko Tamaki, Hiroshi Kusunoki, Yosuke Wada, Shotaro Tsuji, Kana Hashimoto, Takara Mori, Ken Shinmura

**Affiliations:** ^1^ Department of Physical Therapy School of Rehabilitation, Hyogo Medical University Kobe Japan; ^2^ Research Team for Promoting Independence and Mental Health, Tokyo Metropolitan Institute for Geriatrics and Gerontology Tokyo Japan; ^3^ Department of General Internal Medicine School of Medicine, Hyogo Medical University Nishinomiya Japan; ^4^ Department of Internal Medicine Osaka Dental University Hirakata Japan; ^5^ Roppou Clinic Toyooka Japan; ^6^ Department of Orthopedic Surgery School of Medicine, Hyogo Medical University Nishinomiya Japan; ^7^ School of Pharmacy, Hyogo Medical University Kobe Japan; ^8^ Amagasaki Medical COOP Honden Clinic Amagasaki Japan

**Keywords:** older adults, physical activity, questionnaire

## Abstract

**Aim:**

This study aimed to develop and evaluate the Brief Older Adults' Physical Activity Questionnaire (BOPAQ), which was designed to quickly assess moderate‐to‐vigorous physical activity (MVPA) in community‐dwelling older adults.

**Methods:**

We used a cross‐sectional study design involving 165 older participants. The BOPAQ calculated weekly MVPA duration based on two questions regarding the number of days per week engaged in MVPA and the daily duration of activity. Validity was assessed by correlating the MVPA durations derived from the BOPAQ with those obtained from the ActiGraph and International Physical Activity Questionnaire short form. Reliability was evaluated using the intraclass correlation coefficient, and measurement errors were analyzed using Bland–Altman plots.

**Results:**

The BOPAQ reasonably correlated with accelerometer‐based MVPA (rho = 0.297) and showed good test–retest reliability (intraclass correlation coefficient of 0.78, 95% CI 0.64–0.87). In contrast, the correlation between the International Physical Activity Questionnaire short form and accelerometer‐based MVPA was poor (rho = 0.139). The cut‐off value for the BOPAQ was set to identify participants engaging in <150 min of objectively measured physical activity per week, corresponding to the 150‐min threshold. However, the area under the curve in the receiver operating characteristic analyses was not significantly high (0.601, 95% CI 0.514–0.688). The Bland–Altman plots showed an underestimation bias of 51.72 min/week (95% CI 1.61–101.84) and showed heteroscedasticity.

**Conclusion:**

Despite some measurement errors, the BOPAQ is an available tool for assessing MVPA in community‐dwelling older adults. **Geriatr Gerontol Int 2024; 24: 1150–1155**.

## Graphical abstract

We developed the Brief Older Adults' Physical Activity Questionnaire, designed to quickly assess moderate‐to‐vigorous physical activity in community‐dwelling older adults. The Brief Older Adults' Physical Activity Questionnaire showed good reliability and acceptable validity, indicating that it is a useful tool for assessing physical activity in this population.

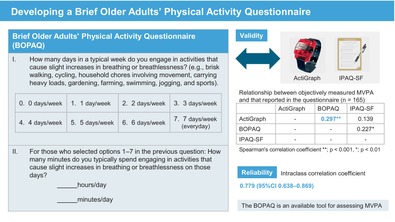

## Introduction

Maintaining a high level of physical activity (PA) and an active lifestyle are key factors in reducing the risk of frailty,^1^ cognitive impairment^2^ and mortality^3^ in older adults. The World Health Organization (WHO) recommends that older adults engage in at least 150 min of moderate‐to‐vigorous PA (MVPA) weekly.^4^ Evaluating PA among older adults is crucial for identifying those who are physically inactive and managing intervention.^5^ In assessing PA, there are methods for objective measurement, as well as methods based on self‐reporting through questionnaires. Although accelerometers, a common method for objectively measuring PA, provide relatively accurate assessments, their availability is limited by the cost of the devices.^6^ Meanwhile, PA questionnaires do not require specific devices and, thus, incur no additional costs. However, existing PA questionnaires, such as the International Physical Activity Questionnaire (IPAQ)^7^ and Physical Activity Scale for the Elderly,^8^ are time‐consuming, limiting their practical use in time‐constrained clinical and community settings.

PA‐related questions in the Japanese version of the Cardiovascular Health Study criteria, which inquire about exercise habits, provide a quick and straightforward method for assessing declines in PA among older adults, and are widely used in clinical and community settings.^9^ A recent study showed that the decline in PA, as determined by the Japanese version of the Cardiovascular Health Study criteria, did not necessarily reflect a decline in accelerometer‐assessed PA.^10^ Additionally, the Japanese version of the Cardiovascular Health Study criteria only assess the presence or absence of exercise habits, which do not allow the quantification of PA levels. However, a valid and quick alternative PA questionnaire to the existing one is currently unavailable.

The present study aimed to develop and evaluate a brief and simple PA questionnaire for older adults.

## Methods

### Study design and participants

We used a cross‐sectional design and collected data from June to November 2023. Community‐dwelling older adults were recruited as a part of the Frail Elderly in Sasayama‐Tamba Area (FESTA) study. The inclusion criteria were as follows: (i) aged ≥65 years, and (ii) ability to walk independently without walking aids, such as walkers. The exclusion criteria were as follows: (i) moderate‐to‐severe cognitive impairment according to a Mini‐Mental State Examination score of <21,^11^ (ii) cardiovascular or pulmonary disease, and (iii) incomplete data.

### Patient consent statement

Before starting the study, the participants were informed about the research both verbally and in writing, and they gave their written consent.

### Development of the brief older adults' physical activity questionnaire

In developing the Brief Older Adults' Physical Activity Questionnaire (BOPAQ), priority was given to creating a questionnaire that could be widely used in clinical and community settings, aiming to produce a tool that allows for quicker and easier completion than existing questionnaires. In line with the WHO recommendations to ensure time for MVPA, the goal was to estimate the total weekly duration of PA exceeding 3 metabolic equivalent of task (METs). Therefore, we utilized question structures from the Health Information National Trends Survey^12^ used in the USA and the Physical Activity Vital Sign developed by the American College of Sports Medicine.^13^ Based on these models, we formulated two items: one to assess the number of days per week engaged in moderate‐to‐vigorous PA and the other to determine the average duration of such activities per day.

The questionnaire was created by three experts: an exercise epidemiologist, a physical therapist specializing in health promotion for older adults and a geriatrician. For the assessment of MVPA, terms, such as “moderate intensity” and “vigorous intensity”, were avoided to ensure clarity for older adults. Activities were defined as those that cause a slight increase in breathing or breathlessness. To better illustrate the types of activities that older adults might engage in, multiple examples of activities anticipated to exceed 3 METs were provided. Normal‐paced walking was not included among these examples, as it might not reach 3 METs in older adults.^14^, ^15^ To reduce omissions in reporting the number of days, eight options were provided, allowing respondents to select the number corresponding to their weekly activity days, with “0” as an option for no MVPA for weeks. The assessment of PA time involved asking the average daily duration of an activity. In accordance with the American PA guidelines in 2018^16^ and the WHO 2020 PA guidelines,^4^ we did not impose the 10‐min bout restriction, allowing for the capture of all periods of activity, regardless of their duration. By reintroducing descriptions related to breathing into the question, we emphasized that the activities were limited to moderate‐to‐vigorous intensity. Taking these considerations into account, the questions were structured as follows: (i) “How many days in a typical week do you engage in activities that cause slight increases in breathing or breathlessness?” (e.g. brisk walking, cycling, household chores involving movement, carrying heavy loads, gardening, farming, swimming, jogging and sports); and (ii) for those who selected options 1–7 in the previous question: “How many minutes do you typically spend engaging in activities that cause slight increases in breathing or breathlessness on those days?” The weekly duration of the MPVA was estimated by combining these two questions.

During the measurement session of our cohort study, we distributed questionnaires to the participants and requested their responses. No additional verbal explanations were provided beyond the written instructions. To investigate the reliability of the questionnaire, we randomly selected approximately 50 participants and mailed the questionnaire to their homes 1 week later for a second response.

### Physical activity assessment

For the objective assessment of PA, we used the ActiGraph wGT3X‐BT (ActiGraph, LLC, Pensacola, FL, USA). The devices were distributed, and the measurement was initiated within 2 weeks of carrying out the questionnaire assessments. The participants were instructed to wear the device on their non‐dominant wrist for seven consecutive days, except during bathing. The sampling frequency was set to 30 Hz. For analysis, the data required at least four valid days, with a valid day defined as a minimum wear time of 600 min.^17^ The nonwear time was assessed using a dual‐window method.^18^ To distinguish sleep from activity analysis, bedtime and wake time were manually recorded using diaries. MVPA was determined by minutes exceeding 7500 vector magnitude (VM) counts per min. Light‐intensity activity thresholds were established between 2000 and 7499 VM counts per min.^19^

We used the IPAQ short form (IPAQ‐SF) to assess usual PA for 7 days as a self‐reported assessment, evaluated concurrently with the BOPAQ. The validity and reliability of the IPAQ‐SF in older adults are reported.^20^, ^21^ The IPAQ consists of three different types of PA: vigorous intensity, moderate intensity and walking, which require continuous PA for >10 min. The total amount of time spent on PA was calculated based on the response to each item. The original version of the IPAQ assigned PA intensity as follows: vigorous intensity 8.0, moderate intensity 4.0 and walking 3.3 METs.^7^ Here, we applied the modified version to older Japanese adults; vigorous intensity 5.3, moderate intensity 3.0 and walking 2.5.^22^ The period of >3.0 METs of PA was defined as MVPA time, and MVPA time per week was estimated.

### Other variables

Information regarding comorbidities was collected based on physician‐conducted interviews. Cognitive function was assessed using the Mini‐Mental State Examination.^23^ To determine gait speed, the participants were instructed to walk at their usual pace along a 12‐m pathway. The time required to traverse a 10‐m section was recorded once and used to calculate gait speed. Maximal handgrip strength was measured twice using the dominant hand, with the highest value recorded in kilograms, using a Smedley‐type handheld dynamometer (GRIP‐A; Takei, Niigata, Japan).

### Statistical analysis

Descriptive statistics were used to analyze the demographic attributes of participants and parameters related to PA. To evaluate the criterion validity of the newly developed PAQ, we analyzed the correlation between the objectively measured MVPA time using an accelerometer and the MVPA time calculated through the IPAQ using Spearman's correlation coefficient. In the review of the validity of self‐reported PA measures and objectively assessed PA in adults, a median correlation coefficient of 0.30 has been reported.^24^ Given that a correlation coefficient of 0.30 is interpreted as an acceptable standard,^7^ this study also adopts 0.30 as an acceptable and usable level. To identify participants with <150 min of MVPA per week (as recommended by the WHO), and determine the cut‐off values, sensitivity and specificity of the new PAQ, we utilized the receiver operating characteristic (ROC) curve. The Bland–Altman plot was used to assess the systematic and random errors between PA measurements from the BOPAQ and accelerometer, with the 95% limits of agreement shown. Furthermore, the test–retest reliability of the new PAQ was assessed by calculating the intraclass correlation coefficient. Following previous research, we evaluated reliability using the criteria that values between 0.5 and 0.75 show moderate reliability, values between 0.75 and 0.9 show good reliability, and >0.90 show excellent reliability.^25^ Data were analyzed using SPSS version 24 (IBM Japan, Tokyo, Japan). Statistical significance was set at *P* < 0.05.

## Results

The present study included a total of 165 participants. The mean age of the participants was 77.0 ± 5.8 years, and 114 of the participants (69%) were women (Table 1). The duration of MVPA per week according to objectively measured PA, BOPAQ and IPAQ were 262 ± 228 min, 210 ± 299 min and 251 ± 440 min, respectively. A total of 103 participants (62.4%) engaged in >150 min of MVPA per week, as recommended by the WHO and as assessed by accelerometers.

**Table 1:** Participants’ characteristics

	*n* = 165
Age, years, mean (SD)	77. 0 (5.8)
Woman, *n* (%)	114 (69)
Height, cm, mean (SD)	154.9 (8.1)
Bodyweight, kg, mean (SD)	54.0 (9.4)
BMI, kg/m^2^, mean (SD)	22.3 (2.7)
Comorbidities	
Hypertension, *n* (%)	73 (44.2)
Dyslipidemia, *n* (%)	42 (25.5)
Diabetes, *n* (%)	14 (8.5)
Kidney disease, *n* (%)	3 (1.8)
Osteoporosis, *n* (%)	19 (11.5)
Cancer, *n* (%)	11 (6.7)
MMSE, median (IQR)	29 (28–30)
Gait speed, m/s (SD)	1.3 (0.2)
Grip strength, kg (SD)	25.8 (6.4)
Objectively measured PA	
Moderate‐to‐vigorous, min/week (SD)	261.4 (217.6)
Light intensity, min/week (SD)	2921.6 (675.4)
Wear time excluding sleep, min/day (SD)	959.6 (73.8)
BOPAQ	
Moderate‐to‐vigorous, min/week (SD)	210 (299)
IPAQ‐SF	
Moderate‐to‐vigorous, min/week (SD)	251.4 (440.1)

BOPAQ, Brief Older Adults' Physical Activity Questionnaire; IPAQ‐SF, International Physical Activity Questionnaire short‐version; IQR, interquartile range; MMSE, Mini‐Mental State Examination; PA, physical activity; SD, standard deviation.

**Figure 1:**
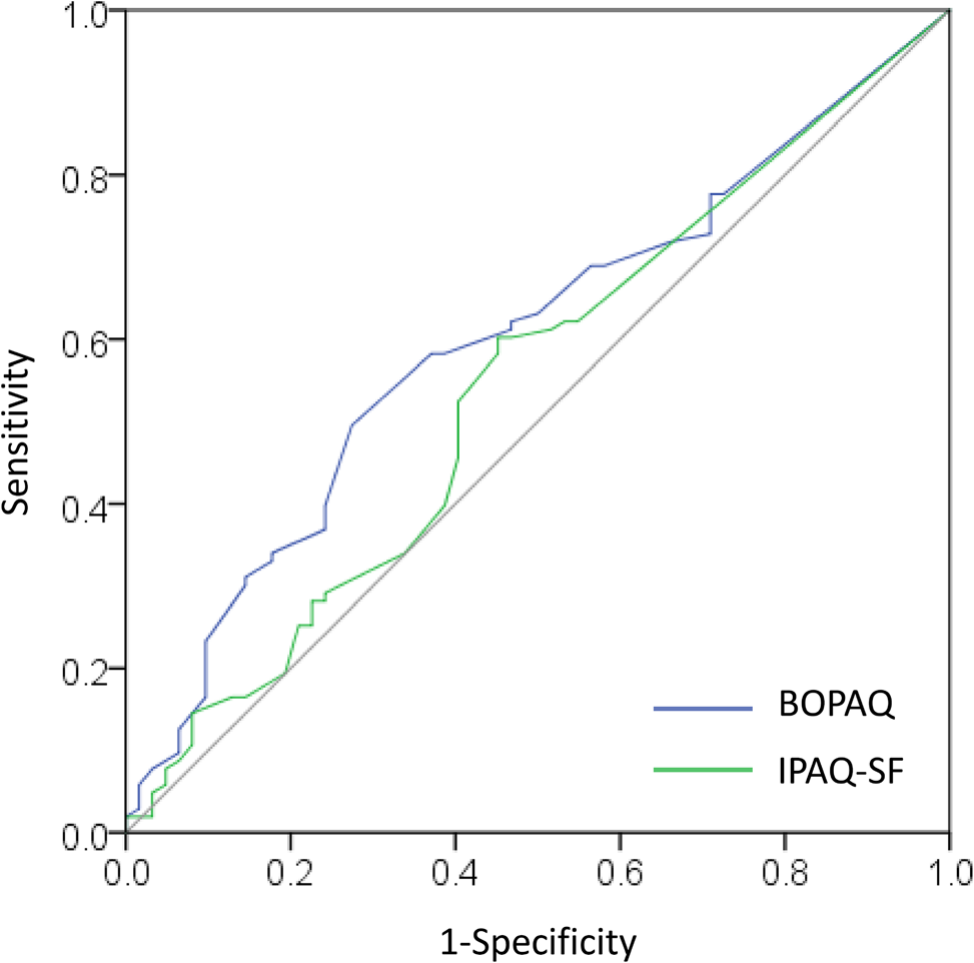
Receiver operating characteristic curves for the Brief Older Adults' Physical Activity Questionnaire (BOPAQ) and the International Physical Activity Questionnaire short form (IPAQ‐SF) to identify whether objectively measured moderate‐to‐vigorous physical activity is <150 min/week. The area under the curve in BOPAQ and IPAQ‐SF were 0.601 (95% CI 0.514–0.688, *P* = 0.030) and 0.543 (95% CI 0.451–0.634, *P* = 0.359), respectively.

The relationships between objectively measured PA and those reported in the questionnaires are presented in Table 2. The duration of MVPA assessed by the BOPAQ was significantly correlated with that assessed by the ActiGraph (rho = 0.297, *P* < 0.001). However, MVPA assessed using the IPAQ‐SF was not (rho = 0.139, *P* = 0.076). The BOPAQ was also significantly correlated with the IPAQ‐SF (rho = 0.227, *P* = 0.003). The area under the curve in the ROC analyses for the BOPAQ and IPAQ‐SF for detection of <150 min of MVPA was 0.601 (95% CI 0.514–0.688, *P* = 0.030) and 0.543 (95% CI 0.451–0.634, *P* = 0.359), respectively (Fig. 1). The cut‐off values for the BOPAQ and IPAQ‐SF were 150 min (sensitivity 0.495, specificity 0.726) and 40 min (sensitivity 0.602, specificity 0.548), respectively.

**Table 2:** Relationship between objectively measured moderate‐to‐vigorous physical activity and that reported in the questionnaire (*n* = 165)

	ActiGraph	BOPAQ	IPAQ‐SF
ActiGraph	‐	0.297**	0.139
BOPAQ	‐	‐	0.227*
IPAQ‐SF	‐	‐	‐

**P < 0.001; *P < 0.01. BOPAQ, Brief Older Adults' Physical Activity Questionnaire; IPAQ‐SF, International Physical Activity Questionnaire short‐version.

**Figure 2:**
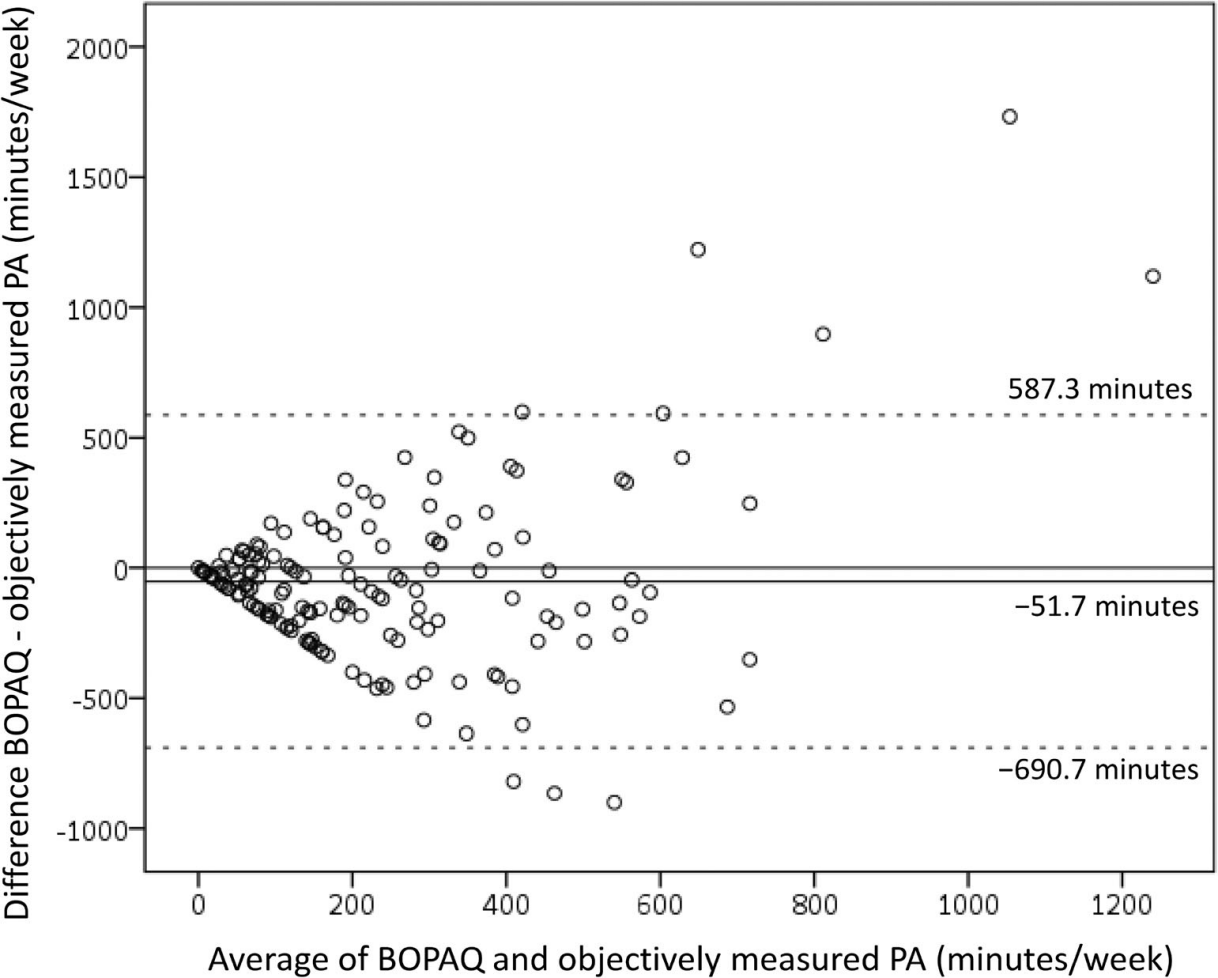
Bland–Altman plot for min/week Brief Older Adults' Physical Activity Questionnaire (BOPAQ) and objectively measured moderate‐to‐vigorous physical activity. The mean error is represented by a solid horizontal line, and the limits of agreement (±1.96 SD from the mean) are shown by dashed horizontal lines. PA, physical activity; SD, standard deviation.

The Bland–Altman plot showed a fixed bias of 51.72 min/week (95% CI 1.61–101.84), indicating that the BOPAQ significantly underestimated MVPA duration compared with accelerometer‐based measurements (Fig. 2). The limits of agreement ranged from −690.7 to 587.3 min/week. Although there was no significant correlation between the difference and mean of the two variables (rho = 0.045, *P* = 0.565), the distribution showed a fan‐shaped spread. This heteroscedasticity suggests that the variability in the differences increases with the magnitude of the measurements. Among the participants with higher mean values, several cases were identified in which the MVPA time reported in the questionnaire was significantly higher than that measured by the accelerometer. The intraclass correlation coefficient for test–retest reliability was 0.779 (95% CI 0.638–0.869), showing good reliability.^25^

## Discussion

In the present study, we developed a brief and simple PA questionnaire for older adults, and evaluated its reliability and validity. The results showed that the newly developed questionnaire demonstrated a weak correlation with MVPA, as objectively measured by accelerometers, indicating acceptable validity. The cut‐off value for the BOPAQ, identifying participants with <150 min/week of objectively measured PA (below the WHO recommendation), matched the 150‐min threshold. However, the ROC values were not particularly high. The BOPAQ tended to underestimate the accelerometer‐measured PA, as measured objectively, with a proportional error that increased with higher levels of measured activity. Nevertheless, its reliability was found to be good.

In the present study, the widely used IPAQ‐SF showed only a weak correlation with MVPA, as measured using accelerometers. Some studies have reported that the IPAQ‐SF has some degree of validity for estimating total PA in older adults.^20^, ^22^ However, when examining the duration of MVPA, the correlation coefficient for older individuals, reported as approximately 0.164,^20^ show that the IPAQ‐SF might not consistently offer highly accurate assessments of MVPA duration. Importantly, the IPAQ was developed for individuals aged 18–65 years,^7^ and does not provide specific examples of activities, such as household chores, gardening or farming, which older adults commonly perform. Furthermore, the IPAQ only assesses activities that last longer than 10 min, thereby excluding shorter yet potentially significant durations. In contrast, the newly developed PA questionnaire for the present study, with correlation coefficients of approximately 0.3, still shows a fair degree of validity when compared with the results of existing questionnaires.^26^ Similar correlation coefficients (0.33 for the long version and 0.30 for the short version) have been reported for the IPAQ among adults aged <65 years.^7^ In a review of physical activity questionnaires and objective physical activity assessments, the median validity correlations across 19 studies involving adults was 0.27.^27^ It has been interpreted that many of these questionnaires are valid for ranking individuals' behavior. A study published in 2018 also interpreted a correlation coefficient of approximately 0.3 as showing acceptable validity for a questionnaire.^28^ Although the BOPAQ showed modest correlations, it demonstrates acceptable validity for a questionnaire.^7^, ^24^, ^27^, ^28^ The BOPAQ avoids terms, such as “high intensity” or “moderate intensity,” and instead directly includes questions on increased breathing rates. Additionally, it does not limit the activity duration to >10 min, and includes examples, such as brisk walking, gardening and farming, which are commonly undertaken by older adults. This clear and comprehensible questioning format for older adults might have contributed to the observed correlation with MVPA measurements using accelerometers.

The ROC curve analysis showed that the 150‐min cut‐off value for the BOPAQ matched the accelerometer‐measured weekly MVPA duration of <150 min. Although the cut‐off value seemed appropriate, the area under the curve of 0.6 was only marginally higher than that for the IPAQ‐SF, indicating that its discriminative ability was modest. This suggests that using the calculated BOPAQ cut‐off value to directly ascertain the presence or absence of reduced PA might not always be suitable. Considering the results of both the correlation and ROC analyses, the BOPAQ appears to reasonably reflect the actual MVPA of older adults. However, the 150‐min cut‐off should be considered strictly for reference.

The Bland–Altman plot showed a consistent underestimation bias in the BOPAQ, with accelerometer‐measured MVPA underestimated by 51.72 min. Studies assessing MVPA duration using questionnaires have reported a mix of overestimations^20^ and underestimations^29^, ^30^ with no clear trends. It is important to be aware that the BOPAQ might underestimate accelerometer‐measured PA duration. Additionally, the plot distribution showed a fan‐shaped spread, and heteroscedasticity was confirmed, indicating that the error might increase with larger MVPA values. Notably, there were several instances where the difference between the BOPAQ and accelerometer‐measured MVPA exceeded the upper limit of agreement, suggesting that some older individuals might significantly overreport their MVPA duration on the questionnaires. Previous research using the IPAQ for older adults has shown that although older adults tend to underreport their MVPA, this underreporting shifts to overreporting as average values increase,^30^ a phenomenon also observed in some cases with the BOPAQ.

Using a questionnaire to evaluate intervention outcomes might not be sensitive enough to detect small changes in activity levels resulting from interventions, as reported in previous studies.^31^, ^32^ The IPAQ guidelines explicitly state that users should carefully consider the range of domains and types of activities included before using the questionnaire, and they clearly advise against its use in small‐scale intervention studies.^33^ The purpose of the present study was to investigate the validity of the BOPAQ in comparison with an accelerometer, not to assess its responsiveness to changes before and after an intervention. Therefore, at this stage, it might be more appropriate to use the BOPAQ primarily as a screening tool to obtain a general understanding of participants' physical activity levels.

The present study had several limitations. First, as this study involved community‐dwelling older adults, the applicability of the findings on reliability and validity to those in institutional settings or who are hospitalized remains uncertain. Second, the study focused on ambulatory older adults, making the applicability of the results to those using mobility aids, such as walkers or wheelchairs, uncertain. Despite concerns regarding the use of cut‐off values for decision‐making, the criterion validity of the newly developed BOPAQ was partially confirmed. Furthermore, the test–retest reliability was found to be good. Importantly, assessing PA using only two questions offers a significant advantage. Consequently, the BOPAQ appears to be a practical tool for the convenient assessment of PA in older adults. Future research should aim to elucidate the longitudinal associations with health‐related outcomes to establish the utility of the questionnaire for assessing PA among older adults.

The BOPAQ developed in the present study shows good reliability and acceptable validity for assessing MVPA duration in older adults. Although the BOPAQ tends to underestimate PA and shows increased errors with longer MVPA durations, its capacity to evaluate PA using only two questions provides a substantial advantage. The BOPAQ is an available tool for assessing PA in community‐dwelling older adults.

## Disclosure statement

The authors declare no conflict of interest.

## Author contributions

KN, RM, HS and KS were involved in the original conception and design of this study. Data acquisition was conducted by KN, RM, KT, HK, YW, ST, KH, MT and KS. KN conducted the statistical analysis. KN, RM and HS interpreted the data. KN drafted the manuscript. RM, HS, KT, HK, YW, ST, KH, MT and KS critically revised the manuscript for intellectual content. All authors read and approved the final manuscript.

## Ethics statement

The research ethics committee of Hyogo Medical University (No. Rinhi 0342) approved this study, which was conducted in accordance with the principles of the Declaration of Helsinki.

## Data Availability

The data that support the findings of this study are available from the corresponding author upon reasonable request.
